# Transmission and Long-term Colonization Patterns of *Staphylococcus aureus* in a Nursing Home

**DOI:** 10.3390/ijerph17218073

**Published:** 2020-11-02

**Authors:** Martyna Kasela, Agnieszka Grzegorczyk, Izabela Korona-Głowniak, Mateusz Ossowski, Bożena Nowakowicz-Dębek, Anna Malm

**Affiliations:** 1 Department of Pharmaceutical Microbiology, Medical University of Lublin, 20-093 Lublin, Poland; agnieszka.grzegorczyk@umlub.pl (A.G.); iza.glowniak@umlub.pl (I.K.-G.); anna.malm@umlub.pl (A.M.); 2 Department of Animal Hygiene and Environmental Hazards, University of Life Sciences in Lublin, 20-950 Lublin, Poland; mateusz.ossowski@up.lublin.pl (M.O.); bozena.nowakowicz@up.lublin.pl (B.N.-D.)

**Keywords:** *Staphylococcus aureus*, transmission, nursing home

## Abstract

Nursing homes might create an environment favorable for the transmission of *Staphylococcus aureus* because of the presence of hospitalized elderly, overcrowding and close contacts among people. We aimed at identifying risk factors for *S. aureus* colonization and determining the genetic relatedness of isolates demonstrating transmission among people. We investigated 736 swab samples from 92 residents and personnel for the presence of *S. aureus*. Swabs from anterior nares and throat were collected quarterly (2018) in a nursing home located in Poland. Genotyping was conducted using the multi-locus variable number of tandem repeats fingerprinting (MLVF) method. We observed high seasonal variation in the proportion of participants colonized with methicillin-resistant *Staphylococcus aureus* (MRSA) strains (0% to 13.5%). A multivariate analysis revealed that residents aged more than 85 years old are at risk for becoming intermittent *S. aureus* carriers (*p* = 0.013). The MLVF analysis revealed a high genetic diversity among methicillin-sensitive *S. aureus* (MSSA) strains and close genetic relatedness between MRSA strains. We proved the advanced aged were predisposed to intermittent *S. aureus* carriage. Genotyping revealed the transmission of *S. aureus* among the participants living in a closed environment. A high genetic relatedness among isolated MRSA suggests its clonal spread in the nursing home.

## 1. Introduction

Nursing homes for the elderly pose as significant reservoirs for *Staphylococcus aureus* and might play an important role in the spread of this pathogen in closed human populations [1]. Elderly people are especially vulnerable for colonization and subsequent infection with multidrug resistant microorganisms as a result of compromised immune systems, frequent hospitalizations, multiple comorbidities, malnutrition, crowding and others [2,3]. Many studies proved healthcare workers act as a vector for MRSA (methicillin-resistant *Staphylococcus aureus*), leading to its nosocomial spread among patients [4,5]. High-risk activities, leading to the transmission of MRSA in long-term care facilities, include dressing, providing hygiene, changing linens, toileting and transferring the residents [5], which suggests not only the nursing personnel is involved in the spread of *S. aureus* but, also, the employees of other professions like physiotherapists, housekeeping staff or eldercare workers. The most significant reservoirs of *S. aureus* among humankind are nares, skin and the gastrointestinal tract [6]. Molecular typing of *S. aureus* collected from different body sites revealed that one person is often colonized by several different strains [1,7]. Sampling multiple body sites in the epidemiological studies increases the detection capabilities of culture methods. The persistence of *S. aureus* carriage is also infrequently studied [1]. It is still unclear why an individual becomes persistently colonized with *S. aureus* or why a carrier acquires new, genetically different strains permanently or transiently. Despite multiple potential factors driving the niche specificity of a strain and its colonization preferences, little is known about *S. aureus* ecology as an opportunistic microorganism [8]. The colonization and infection rates for MSSA (methicillin-susceptible *Staphylococcus aureus*) and MRSA strains remain highly diversified worldwide and depend on the demographic characteristics of the studied population, as well as the geographical location [9]. To our best knowledge, there is no epidemiological data concerning *S. aureus* colonization rates in nursing homes located in Poland.

The aim of our study was to investigate the local epidemiological situation in a nursing home located in Eastern Poland (Lublin) by determining the longitudinal *S. aureus* colonization patterns, identifying potential risk factors predisposed to MSSA and MRSA carriage and establishing the genetic relatedness of isolated *S. aureus* strains using the MLVF (multi-locus variable number of tandem repeats fingerprinting) method. We asked whether there are factors influencing the duration of *S. aureus* colonization and tested the hypothesis that high genetic relatedness indicates the transmission of *S. aureus* in nursing home residents and personnel.

## 2. Materials and Methods

### 2.1. Study Design and Sample Collection

**Figure 1:**
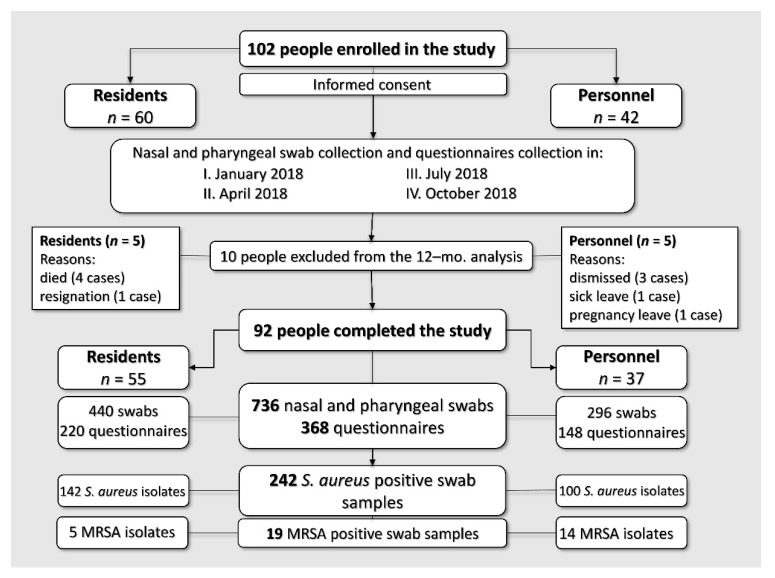
Participant enrollment scheme. MRSA: methicillin-resistant *Staphylococcus aureus*.

The retrospective and longitudinal study was carried out in order to evaluate factors that might be associated with the colonization and carriage of MSSA and MRSA among the residents and personnel of a nursing home, as well as to determine the phylogenetic relationship of *S. aureus* strains, indicating the interparticipant *S. aureus* transmission. A total number of 102 people participated in our study; however, only 92 of them were present in all four sampling periods and, thus, were enrolled into the final analysis (Figure 1). We collected the swabs from the anterior nares and the throats of the participants in January, April, July and October 2018 (once every three months). The samples were never taken during antibiotic therapy. Swabs were collected at least 14 days after the last dose of antibiotic was administered or before antibiotic therapy, when it was possible. During days when samples were collected, the questionnaires in a paper form were self-administered for all personnel members and for residents able to write. For residents not able to write, data were collected from medical documentation. We collected the following information: age, gender, smoking tobacco products, the number of antibiotics administered, the number of hospitalizations during the sampling period (starting from 3 months before the first sampling period) and the occurrence of underlying diseases. Additional questions for personnel were the duration of work and current profession in a nursing home. Based on the information obtained in the questionnaires, personnel members were divided on two groups according to the occurrence of close contacts with the residents or their personal belongings during daily work. The personnel members of all the professions were involved in the study (nurses, housekeeping staff, physiotherapists, eldercare workers, laundresses, cooks and administration staff).

Two anatomical locations—the anterior nares and the throat—were swabbed using sterile cotton swabs (Medlab, Warszawa, Poland) premoistened with 0.9% saline solution (POCH, Gliwice, Poland). The samples were immediately transported to the laboratory and plated on tryptic soy agar (TSA; BioRad, CA, USA) with 5% of sheep blood and mannitol salt agar (BioMaxima, Lublin, Poland) and incubated for 24–48 h at 35 °C. The colonization of the upper respiratory tract by a relatively small number of *S. aureus* cells may negatively affect the detection capabilities of culture methods based on the swab collection. Therefore, we defined persistent carriage as the presence of *S. aureus*-positive swab samples in a certain site (anterior nares or throat or both sites at the same time) during at least three out of four sampling periods. Other participants colonized during the study were classified as intermittent carriers.

### 2.2. S. aureus Identification and Detection of Methicillin-resistance

Species identification was performed using the Vitek 2 Compact automated system and GP cards (Biomerieux, Marcy l’Etoile, France). *S. aureus* methicillin resistance was detected by the disc-diffusion method using cefoxitin discs (Becton Dickinson, New Jersey, NJ, USA), as recommended by EUCAST (version 8.1). *S. aureus* isolates were stored in freezers (−70 °C) for further analysis using TSB (tryptic soy broth; BTL, Warszawa, Poland) containing 50% glycerol (POCH, Gliwice, Poland).

### 2.3. Multi-Locus Variable Number of Tandem Repeats Fingerprinting (MLVF)

Bacterial DNA was extracted from fresh (24-h) *S. aureus* cultures in TSB, according to the manufacturer’s instructions (Genomic Mini; A&A Biotechnology, Gdynia, Poland). Genotyping of 242 *S. aureus* strains by multi-locus variable number of tandem repeats fingerprinting was conducted using primers proposed elsewhere [10] and a modified PCR program [11] (Whatman; Biometra, Maidstone, UK). The PCR products and DNA molecular size marker (100-bp DNA Ladder Plus; Thermo Scientific, MA, USA) were loaded into 2% agarose gel (Sigma-Aldrich, MO, USA) stained with SimplySafe dye (EURx, Gdańsk, Poland). The products were separated at a constant voltage of 120 V. Gels were photographed, and the dendrogram was constructed by applying the UPGMA (unweighted pair-group method with arithmetic mean) algorithm and 1% tolerance (BioGene; Polygen, Gliwice, Poland).

### 2.4. Statistical Analysis

Data processing and analysis were performed using Tibco Statistica Ver. 13.3 (TibcoSoft. Inc., Palo Alto, CA, USA). The results were expressed as percentage or mean with standard deviation (SD). In univariate analyses, the following risk factors were investigated: age—median (range) in personnel group and resident group were divided in two groups: aged >85 and ≤85; gender; smoking tobacco products (yes or no); the number of antibiotics administered prior to the sampling period (none, 1 antibiotic and ≥2 antibiotics); the number of hospitalizations during the 12-month sampling period (starting from 3 months before the first sampling period: none, 1 stay and ≥2 stays) and the occurrence (yes or no) of underlying diseases (diabetes mellitus, cancer renal disease, neurological disorders, respiratory tract disorders, cardiovascular disease or immunosuppressive drug medication). The analyses were performed using chi-square or Fisher’s exact test, depending on the size of samples and of contingency tables for categorical variables and using the Mann-Whitney U test for continuous variables (age in median). Odds ratios (OR) and their 95% confidence intervals (CI) were calculated. Statistical significance was set if the 2-tailed *p*-value was <0.05.

### 2.5. Ethical Statement

This study was approved by the Bioethics Committee at the Medical University of Lublin, Poland (KE 0254/59/2016). Written informed consent was obtained from each individual before participation.

## 3. Results

The mean age of the residents (80 ± 9.8 years old) was almost twice as high as that of the personnel (42.7 ± 9.1 years old) (Table 1). We observed the prevalence of females in residents (70.9%) and personnel (81.1%). Despite the frequency of hospitalization was higher in residents (29.1%) than in personnel (2.7%), the average number of antibiotic courses among both groups were similar: over one-third of the residents (34.5%) and personnel members (35.1%) had at least one antibiotic therapy during the study period. Cardiovascular diseases were the most prevalent comorbidities among the residents of a nursing home (85.5%).

### 3.1. S. aureus Carriage among the Residents and Personnel of a Nursing Home

Among all participants included into the analysis, only 30.9% (17/55) of residents and 16.2% (6/37) of personnel members tested negative for the presence of *S. aureus* in the upper respiratory tract. *S. aureus* was isolated at least once from 69.1% (38/55) residents and 83.8% (31/37) personnel members (OR 2.3, 95% Cl 0.8–6.6, *p* = 0.14). The rates of persistent and intermittent carriers in residents accounted for 34.5% each. In the personnel group, 40.5% of people were classified as persistent carriers and 43.2% as intermittent. There was no statistically significant differences between the residents and the personnel in the percentage of persistent (OR 0.77, 95% Cl 0.3–1.8, *p* = 0.66) and intermittent *S. aureus* colonization (OR 0.69, 95% Cl 0.3–1.6, *p* = 0.51). The most prevalent anatomical locations in residents that was persistently colonized with *S. aureus* were the anterior nares and throat at the same time (42.1%), followed by anterior nares (36.8%) and throat (21.2%). *S. aureus* niche preference in the personnel group differed. The anterior nares were the most often inhabited body site (53.3%), then the throat (26.7%) and the anterior nares and throat simultaneously (20%).

### 3.2. MRSA Colonization among the Residents and Personnel of a Nursing Home

In total, we isolated 19 MRSA strains from 10 participants—three residents and seven personnel members. The prevalence of MRSA colonization varied in different seasons of the year. Winter was the only period of the year in which we did not detect any MRSA isolates. Low MRSA colonization rates among the residents and personnel were observed during the spring and accounted for 0% and 5.4%, respectively. MRSA was isolated from 1.8% residents in the summer and 3.6% in the autumn. The highest MRSA colonization rates were noted for the personnel, increasing to 13.5% in the summer and 8.1% in the autumn.

### 3.3. Risk Factors for S. aureus Carriage among the Residents and Personnel of a Nursing Home

Statistical analysis revealed that residents aged more than 85 years old are at-risk for becoming intermittent S. aureus carriers (*p* = 0.02). None of the other factors taken into account were significantly associated with S. aureus carriage and its type (Table 2). Personnel members with underlying diseases were more likely to carry S. aureus in their upper respiratory tract; however, the association was not statistically significant (*p* = 0.065). We observed that the duration of S. aureus carriage differed in people who were undergoing antibiotic therapy (Table 3). Not taking the antibiotics showed a predisposition to the intermittent type of S. aureus carriage in personnel members (*p* = 0.019).

### 3.4. Risk Factors for MRSA Colonization

Although we found no significant risk factors for MRSA colonization among all participants, females (*p* = 0.061) and the personnel members (*p* = 0.083) were more likely to be colonized with MRSA strains (Table 4). The analogous situation was found exclusively among *S. aureus* carriers, where females (*p* = 0.053) were more often colonized with the methicillin-resistant strains of *S. aureus* (Table 5).

### 3.5. Genotyping of S. aureus by Multi-Locus Variable Number of Tandem Repeats Fingerprinting

We analyzed the genetic profiles of all collected *S. aureus* strains: 225 MSSA and 19 MRSA. The dendrogram is presented in Figure S1 (supplement). Genotyping of the 244 *S. aureus* isolates by MLVF identified 84 different banding patterns (Figure 1).Among them, 34 (40.5%) were represented by one *S. aureus* strain only, 22 (26.1%) were represented by two to three strains and 28 (33.3%) were represented by four to eight isolates. Moreover, seven (8.3%) banding patterns consisted of *S. aureus* isolated from two or three different individuals. Genotyping grouped 244 *S. aureus* isolates (100%) into two main clusters (cut-off > 70%). The main cluster A contained 203 *S. aureus* isolates (83.2%) and was divided into cluster A1 (156 isolates) and cluster A2 (47 isolates). The main cluster B, which was much smaller, contained 41 *S. aureus* isolates (16.8%) and was divided into clusters: B1 (25 isolates) and B2 (16 isolates). In contradistinction to the high genetic diversity of MSSA strains, MRSA strains were characterized by a high genetic similarity. As much as six MRSA (31.6%) strains from three different individuals were characterized by the same MLVF banding pattern and, additionally, were clustered altogether with four MRSA isolated from other three persons.

Among persistent carriers simultaneously colonized in the anterior nares and throat, 81.8% (9/11) carried the same *S. aureus* MLVF type in both sampling sites. The MLVF genotypes of *S. aureus* in people persistently colonized remained relatively stable over time, which means all strains isolated from an individual during the study had 100% homology, with a minor exception when single strains were related at a level higher than 80%. Only 20.6% (7/34) of persistent carriers were characterized by the presence of single genetically unrelated *S. aureus* strains at some point of the study, which also concerned the temporal colonization with MRSA.

## 4. Discussion

Antibiotic therapy disrupts the homeostasis between the host and the physiological components of the microbiota [12,13]. We found that over one-third of the participants had at least one antibiotic therapy during the 12-month study period. The host deprived of the protective role of commensal microorganisms is more vulnerable for colonization with potentially pathogenic bacteria. Rondeau et al. (2016) found that the antibiotic therapy and health status of the nursing home residents were significantly associated with MRSA colonization [14]. The adverse effects of the use of antibiotics are also well-known for their contribution to the development of the acquired antibiotic resistance among microorganisms [15]. We observed that the type of *S. aureus* carriage (persistent or intermittent) varied among the personnel members that underwent antibiotic therapy (*p* = 0.019). The effects that perturbations like antibiotic therapy have on the original composition of protective microbiota in the respiratory tract are multifaceted and might be relevant for the colonization and carriage of potentially pathogenic bacterial species [16].

The statistical analysis revealed that ages over 85 years old were a significant risk factor for *S. aureus* intermittent carriage among the residents (*p* = 0.02). Advanced age was previously considered by other authors as a risk factor for higher morbidity among nursing home residents with staphylococcal infection [17] and for the development of community onset pneumoniae caused by MRSA [18]. Poor health status, multiple comorbidities and malnutrition lead to the dysfunction of the host’s immune system [2]. Progressive imbalance between the host and microbiota, as well as between particular microbiota components, enables new microorganisms from the environment to colonize the skin and mucous membranes and, consequently, to compete for the ecological niche. This would explain our findings about the intermittent character of *S. aureus* carriage of the upper respiratory tract among the elderly. Although the issue is not the main subject of our research, further studies should focus on evaluating the association between the resilience of the host’s microbiota of the upper respiratory tract and the susceptiveness on MRSA colonization [16].

Frequent hospitalizations among the residents might lead to the transmission of nosocomial pathogens to the nursing home environment, implying an increased risk of colonization with antibiotic-resistant microorganisms among the elderly with weakened immune systems. Hospitals are a source of MRSA and an important route of its transmission to long-term care facilities [19]. Large-scale studies conducted in Germany showed that the rates of MRSA colonization among the healthcare personnel are usually higher in the nosocomial environment (4.6%) than in the facilities for outpatient geriatric and nursing care (1.2%) [20,21]. Nevertheless, sharing the patients between nursing homes and hospitals affects the rates of MRSA colonization in both types of facilities [22].

We discovered the seasonal fluctuation of MRSA colonization rates in the personnel group (0–13.5%). Higher MRSA colonization rates during the summer were also observed in other studies [23]. This finding suggests that MRSA prevalence in nursing homes varies not only between different countries and facilities [24,25] but, also, within certain care centers. Pathare et al. (2016) noticed the high nasal MRSA colonization rates among the personnel of a nursing home (15.1%), as well as a significant contamination of their cell phones (9%) [4]. However, contrary to our results, most of the studies indicated that MRSA colonization rates were higher in residents than the personnel of nursing homes [26]. Environmental MRSA contamination was significantly associated with its intrahousehold transmission [27] and might affect the transmission levels in nursing homes [28]. According to the Chamchod and Ruan (2012) model of MRSA spread in nursing homes, the most effective control measures include screening at admission, decolonization, proper hand hygiene and increasing the ratio between personnel and the residents [29]. The effectiveness of the so-called “search and destroy” policy implemented by the Scandinavian countries has been confirmed by low MRSA rates presented in many studies [30,31].

The mutation frequency of *S. aureus* in different conditions remains unknown, making the determination of epidemiological relatedness difficult. The intricateness of this issue is reasserted by multiple factors influencing the mutation frequency: the type of the ecological niche being colonized, *S. aureus* interaction with other bacterial species and the host’s immune system, transfer to a new host, the presence of disinfectants and antibiotics in the environment and many others [32]. We chose the MLVF method because it is well-known for its high discriminatory potential and suitability for the investigation of local transmission patterns [11]. We observed the presence of single genetically unrelated strains in persistent *S. aureus* carriers at some point over the 12 months of study. The situation was probably caused by simultaneous colonization with multiple genetically unrelated strains rather than temporal strain replacement. The so-called “co-colonization” of different *S. aureus* genotypes was previously observed in many studies, which often included the presence of mixed colonization with MSSA and MRSA at one time [7,33]. The data obtained with MLVF typing showed that the *S. aureus* population structure among the residents and personnel of a nursing home is highly diversified and dynamically changes over time. The presence of multiple identical genetic profiles shared by two or even three persons suggests the continuous process of *S. aureus* transmission among people living and working in a nursing home. A high genetic similarity of MRSA strains isolated from the personnel of a nursing home indicates the possibility of its clonal spread, as well as underlines the personnel role as a MRSA vector.

## 5. Conclusions

Acquiring and loosing *S. aureus* strains by carriers, as well as its transmission among people, is becoming more complex and leaves a lot of unanswered questions. We found that personnel members were more often colonized by *S. aureus* than the residents, and the duration of colonization depended on the occurrence of antibiotic therapy. The residents aged more than 85 years old were at high risk for becoming intermittent *S. aureus* carriers, which could have meaning in the context of *S. aureus* transmission in a closed population. The prevalence of MRSA colonization varied in different seasons of the year (0–13.5%), and a high genetic relatedness suggested its clonal spread in the nursing home.

## Figures and Tables

**Table 1:** Demographic and clinical characteristics of the participants.

Variable	Total *n* = 92	Residents *n* = 55	Personnel *n* = 37
Age (years), mean ± SD ^1^	65 ± 20.7	80 ± 9.8	42.7 ± 9.1
Female, *n* (%)	69 (75)	39 (70.9)	30 (81.1)
Male, *n* (%)	23 (25)	16 (29.1)	7 (18.9)
Smoking, *n* (%)	13 (14.1)	8 (14.5)	5 (13.5)
Recent antibiotic use, *n* (%)			
0	60 (65.2)	36 (65.5)	24 (64.9)
1	22 (23.9)	11 (20)	11 (29.7)
≥2	6 (6.5)	8 (14.5)	2 (5.4)
Hospital in the past one year, *n* (%)			
0	75 (81.5)	39 (70.9)	36 (96.3)
1	11 (12)	10 (18.2)	1 (2.7)
≥2	6 (6.5)	6 (10.9)	0
Underlying diseases, *n* (%)			
Diabetes mellitus	17 (18.5)	14 (25.5)	3 (8.1)
Cancer	4 (4.3)	2 (3.6)	2 (5.4)
Renal disease	7 (7.6)	7 (12.7)	0
Neurological disorders	19 (20.7)	19 (34.5)	0
Gastrointestinal disease	19 (20.7)	17 (30.9)	2 (5.4)
Respiratory tract disorders	10 (10.9)	9 (16.4)	1 (2.7)
Cardiovascular disease	53 (57.6)	47 (85.5)	6 (16.2)
Immunosuppressive drugs	7 (7.6)	7 (12.7)	0

^1^ SD—Standard deviation.

**Table 2:** Risk factors for *Staphylococcus aureus* carriage among nursing home residents (*n* = 55)

Variable	Univariate Analysis					
	Carriers (Persistent and Intermittent) (*n* = 38)	Noncarriers (*n* = 17)	*p* Value	Persistent Carriers (*n* = 19)	Intermittent Carriers (*n* = 19)	*p* Value
Age (years), mean ± SD	80.3 ± 10.2	79.0 ± 9.3				
<85	22 (57.9)	11 (64.7)	0.77	7 (36.8)	15 (78.9)	0.02
> 85	16 (42.1)	6 (35.3)		12 (63.2)	4 (21.1)	
Female, *n* (%)	27 (71.1)	12 (70.6)	1.0	14 (73.7)	13 (68.4)	1.0
Male, *n* (%)	11 (28.9)	5 (29.4)		5 (26.3)	6 (31.6)	
Smoking, *n* (%)	5 (13.2)	3 (17.7)	0.69	1 (5.3)	7 (21.1)	0.34
Recent antibiotic use, *n* (%)						
0	24 (63.2)	12 (70.6)	0.28	12 (63.2)	12 (63.2)	0.83
1	9 (23.7)	2 (11.8)		5 (26.3)	4 (21.1)	
≥2	5 (13.2)	3 (17.7)		2 (10.5)	3 (15.8)	
Hospitalization in past one year, *n* (%)						
0	27 (71.1)	12 (70.6)	0.22	14 (73.7)	13 (68.4)	0.39
1	5 (13.2)	5 (29.4)		3 (15.8)	2 (10.5)	
≥2	6 (15.8)	0 (0)		2 (10.5)	4 (21.1)	
Underlying diseases, *n* (%)						
Diabetes mellitus	8 (21.1)	6 (35.3)	0.32	2 (10.5)	6 (31.6)	0.23
Cancer	1 (2.6)	1 (5.9)	0.53	0 (0)	1 (5.3)	1.0
Renal disease	4 (10.5)	3 (17.7)	0.66	3 (15.8)	1 (5.3)	0.60
Neurological disorders	12 (31.6)	7 (41.2)	0.55	5 (26.3)	7 (36.8)	0.73
Gastrointestinal disease	9 (23.7)	8 (47.1)	0.12	5 (26.3)	4 (21.1)	1.0
Respiratory tract disorders	6 (15.8)	3 (17.7)	1.0	2 (10.5)	4 (21.1)	0.66
Cardiovascular disease	33 (86.8)	14 (82.4)	0.69	16 (84.2)	17 (89.5)	1.0
Immunosuppressive drugs	3 (7.9)	3 (17.7)	0.36	3 (15.8)	0 (0)	0.23

**Table 3:** Risk factors for *Staphylococcus aureus* carriage among nursing home personnel (*n* = 37).

Variable	Univariate Analysis					
	Carriers (Persistent and Intermittent) (*n* = 31)	Noncarriers (*n* = 6)	*p* Value	Persistent Carriers (*n* = 15)	Intermittent Carriers (*n* = 16)	*p* Value
Age (years), mean ± SD	42.6 ± 9.5	43.0 ± 7.7	0.93	43.3 ± 10.9	42.0 ± 8.2	0.70
Female, *n* (%)	24 (77.4)	6 (100)	0.57	10 (66.7)	14 (87.5)	0.22
Male, *n* (%)	7 (22.6)	0 (0)		5 (33.3)	2 (12.5)	
Smoking, *n* (%)	4 (12.9)	1 (16.7)	1.0	3 (20.0)	1 (6.3)	0.33
Close contact with residents, *n* (%)	22 (71.0)	4 (66.7)	1.0	11 (73.3)	11 (68.8)	1.0
Duration of work (months), mean (range)	84 (12–480)	60 (24–420	0.77	60 (24–252)	78 (12––480)	0.72
Recent antibiotic use, *n* (%)						
0	19 (61.3)	5 (83.3)	0.46	7 (46.7)	12 (75.0)	0.019
1	10 (32.3)	1 (16.7)		8 (53.3)	2 (12.5)	
≥2	2 (6.4)	0 (0)		0 (0)	2 (12.5)	
Hospitalization in past one year, *n* (%)						
0	30 (96.8)	6 (100)	1.0	14 (93.3)	16 (100)	0.48
1	1 (3.2)	0 (0)		1 (6.7)	0 (0)	
Underlying diseases, *n* (%)						
Diabetes mellitus	3 (9.7)	0 (0)	1.0	0 (0)	3 (18.8)	0.22
Cancer	2 (6.5)	0 (0)	1.0	1 (6.7)	1 (6.3)	1.0
Gastrointestinal disease	2 (6.5)	0 (0)	1.0	1 (6.7)	1 (6.3)	1.0
Respiratory tract disorders	1 (3.2)	0 (0)	1.0	0 (0)	1 (6.3)	1.0
Cardiovascular disease	6 (19.4)	0 (0)	0.56	3 (20.0)	3 (18.8)	1.0

**Table 4:** Risk factors for methicillin-resistant *Staphylococcus aureus* (MRSA) carriage among the entire population.

Variable	Univariate Analysis			
	Total (*n* = 92)	MRSA Carriers (*n* = 10)	Others/Noncarriers (*n* = 82)	*p* Value
Age (years), median (range)	81 (56–97)	45 (28–97)	70 (26–94)	0.17
Female, *n* (%)	69 (75.0)	10 (100)	59 (72.0)	0.061
Male, *n* (%)	23 (25.0)	0 (0)	23 (28.0)	
Smoking, *n* (%)	13 (14.1)	1 (10.0)	12 (14.6)	1.0
Nursing home residents, *n* (%)	55 (59.8)	3 (30.0)	52 (63.4)	0.083
Persistent carriage, *n* (%)	34 (37.0)	3 (30.0)	31 (37.8)	0.74
Recent antibiotic use, *n* (%)				
0	60 (65.2)	6 (60.0)	54 (65.9)	0.60
1	22 (23.9)	2 (20.0)	20 (24.4)	
≥2	9 (9.8)	2 (20.0)	7 (8.5)	
Hospitalization in past one year, *n* (%)				
0	75 (81.5)	10 (100)	65 (79.3)	0.23
1	11 (12.0)	0 (0)	11 (13.4)	
≥2	6 (6.5)	0 (0)	6 (7.3)	
Underlying diseases, *n* (%)				
Diabetes mellitus	17 (18.5)	3 (30.0)	14 (17.1)	0.39
Cancer	4 (4.4)	0 (0)	4 (4.9)	1.0
Renal disease	7 (7.6)	1 (10.0)	6 (7.3)	0.57
Neurological disorders	19 (20.7)	1 (10.0)	18 (22.0)	0.68
Gastrointestinal disease	19 (20.7)	3 (30.0)	16 (19.5)	0.43
Respiratory tract disorders	10 (10.9)	2 (20.0)	8 (9.8)	0.30
Cardiovascular disease	53 (57.6)	5 (50.0)	48 (58.5)	0.74
Immunosuppressive drugs	6 (6.5)	1 (10.0)	5 (6.1)	0.51

**Table 5:** Risk factors for MRSA colonization among *Staphylococcus aureus* carriers.

Variable	Univariate Analysis			
	Total *n* = 69	MRSA Carriers *n* = 10	Non-MRSA Carriers *n* = 59	*p* Value
Age (years), median (range)	65 (26–97)	45 (28–97)	68 (26–97)	0.25
Female, *n* (%)	51 (73.9)	10 (100)	41 (69.5)	0.053
Male, *n* (%)	18 (26.1)	0 (0)	18 (30.5)	
Smoking, *n* (%)	9 (13.0)	1 (10.0)	8 (13.6)	1.0
Nursing home residents, *n* (%)	38 (55.1)	3 (30.0)	35 (59.3)	0.10
Persistent carriage, *n* (%)	34 (49.3)	3 (30.0)	31 (52.5)	0.31
Recent antibiotic use, *n* (%)				
0	43 (62.3)	6 (60.0)	37 (62.7)	0.30
1	19 (27.5)	2 (20.0)	17 (28.8)	
≥2	4 (5.8)	2 (20.0)	4 (6.8)	
Hospitalization in past one year, *n* (%)				
0	57 (82.6)	10 (100)	47 (79.7)	0.24
1	6 (8.7)	0 (0)	6 (10.2)	
≥2	6 (8.7)	0 (0)	6 (10.2)	
Underlying diseases, *n* (%)				
Diabetes mellitus	11 (15.9)	3 (30.0)	8 (13.6)	0.19
Cancer	3 (4.4)	0 (0)	3 (5.1)	1.0
Renal disease	4 (5.8)	1 (10.0)	3 (5.1)	0.47
Neurological disorders	12 (17.4)	1 (10.0)	11 (18.6)	0.68
Gastrointestinal disease	11 (15.9)	3 (30.0)	8 (13.6)	0.19
Respiratory tract disorders	7 (10.1)	2 (20.0)	5 (8.5)	0.27
Cardiovascular disease	39 (56.5)	5 (50.0)	34 (57.6)	0.74
Immunosuppressive drugs	3 (4.4)	1 (10.0)	0 (0)	0.38

## Supplementary Materials

The following are available online at https://www.mdpi.com/1660-4601/17/21/8073/s1 Figure S1: Multi-locus variable number of tandem repeats (MLVF) dendrogram of 242 *Staphylococcus aureus* isolates generated by the UPGMA algorithm. The MLVF main clusters are indicated on the right side of the dendrogram. Methicillin-resistant *Staphylococcus aureus* (MRSA) strains were marked red. Isolates names contain information about the number of a person, studied group (RES—residents and PER—personnel), the isolation site (T—throat and N—anterior nares) and the time of isolation (I—January 2018, II—April 2018, III—July 2018 and IV—October 2018), e.g., the isolate named 18–RES–T–I should be read as *S. aureus* isolated from resident no. 18 from the throat in January 2018.
