# Supplementary figures and images for: Transmission and Long-term Colonization Patterns of Staphylococcus aureus in a Nursing Home
Source: Int J Environ Res Public Health. 2020 Nov 2;17(21):8073. doi: 10.3390/ijerph17218073 (PMC7672560; doi:10.3390/ijerph17218073)

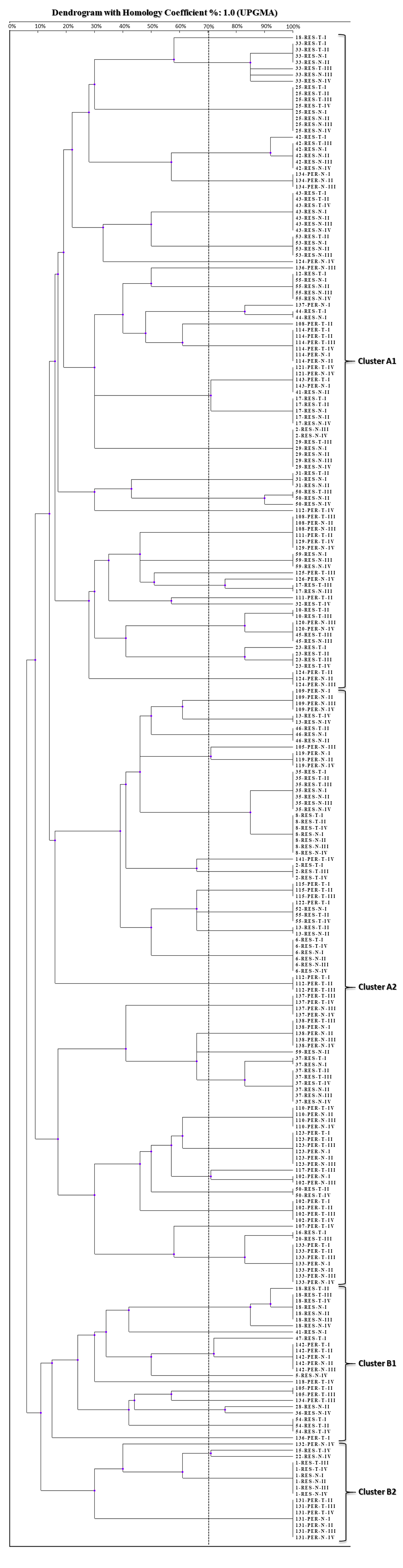

Supplement: Supplementary file 1 [file ijerph-17-08073-s001.bmp]
